# Concurrent profiling of polar metabolites and lipids in human plasma using HILIC-FTMS

**DOI:** 10.1038/srep36490

**Published:** 2016-11-07

**Authors:** Xiaoming Cai, Ruibin Li

**Affiliations:** 1 School of Public Health, Soochow University, Suzhou 215123, China; 2 School for Radiological and Interdisciplinary Sciences (RAD-X), Collaborative Innovation Center of Radiation Medicine of Jiangsu Higher Education Institutions, Soochow University, Suzhou 215123, China; 3 Department of Pharmacology, University of California, Irvine, Irvine, CA 92697, United States; 4 Division of NanoMedicine, Department of Medicine, University of California, Los Angeles, CA 90095, United States

## Abstract

Blood plasma is the most popularly used sample matrix for metabolite profiling studies, which aim to achieve global metabolite profiling and biomarker discovery. However, most of the current studies on plasma metabolite profiling focused on either the polar metabolites or lipids. In this study, a comprehensive analysis approach based on HILIC-FTMS was developed to concurrently examine polar metabolites and lipids. The HILIC-FTMS method was developed using mixed standards of polar metabolites and lipids, the separation efficiency of which is better in HILIC mode than in C5 and C18 reversed phase (RP) chromatography. This method exhibits good reproducibility in retention times (CVs < 3.43%) and high mass accuracy (<3.5 ppm). In addition, we found MeOH/ACN/Acetone (1:1:1, v/v/v) as extraction cocktail could achieve desirable gathering of demanded extracts from plasma samples. We further integrated the MeOH/ACN/Acetone extraction with the HILIC-FTMS method for metabolite profiling and smoking-related biomarker discovery in human plasma samples. Heavy smokers could be successfully distinguished from non smokers by univariate and multivariate statistical analysis of the profiling data, and 62 biomarkers for cigarette smoke were found. These results indicate that our concurrent analysis approach could be potentially used for clinical biomarker discovery, metabolite-based diagnosis, *etc*.

Metabolite profiling is defined as the measurement of low-molecular-weight metabolic analytes and their intermediates that reflect the dynamic response of biological systems to various biological conditions, such as disease and environmental exposure^1^,^2^. These small metabolic analytes are the end products of gene expression or protein activity^3^,^4^, and are composed of a number of chemically diverse compounds including amino acids, lipids, sugars, nucleic acids, simple fatty acids, *etc*^5^. Blood samples are relatively easy to collect, less invasive and more stable, compared to other body fluids. Therefore, metabolite profiling of blood samples has been widely used in biomarker discovery^6^,^7^,^8^,^9^,^10^,^11^ and assessments of adverse outcome pathways (AOPs) induced by chemicals^12^, drugs^13^,^14^,^15^ and environmental stress^16^. Liquid chromatography coupled with mass spectrometry (LC-MS) is one of the main techniques for blood plasma metabolite profiling^17^,^18^,^19^. To date, many of these studies focused on either the polar metabolite^20^,^21^ or lipid fractions^6^,^22^,^23^,^24^. A comprehensive coverage of metabolites, however, is important when examining the biochemical activities in an organism because both polar metabolites and lipids in multiple metabolism pathways can often be simultaneously influenced by a single biological event, such as smoking^25^, inflammation^26^ and diabetes^27^. A few studies that refer to global plasma/serum metabolite profiling usually required dual extraction and/or dual separation^27^,^28^,^29^,^30^,^31^, which are time consuming. Therefore, it’s necessary to develop a single extraction-single separation approach for concurrent analysis of both polar metabolites and lipids in blood plasma.

Metabolite profiling of blood plasma demands an efficient extraction approach, which usually requires sufficient deproteinization, high solubilization of metabolites and minimum sample handling process. Organic solvents (such as methanol, acetonitrile, ethanol, acetone and their mixtures) have been proven to be useful for metabolite solubilization and protein precipitation^32^,^33^,^34^. In addition, biphasic solvents, which separate the plasma metabolites into polar and non-polar fractions, are widely used^29^,^31^,^35^. Although organic solvents have shown many advantages on metabolite extraction, there is not a universal solvent receipt for high efficient extraction because of the differences in samples and analysis methods. Therefore, it is necessary to develop a cocktail of extraction solvents for simultaneous analysis of polar metabolites and lipids in plasma.

An efficient LC separation prior to MS detection is also very important for the analysis of metabolites in complex biological samples^36^. Reversed phase liquid chromatography (RPLC) is the most popularly used separation method^18^,^19^. However, polar and ionic metabolites, such as organic acids and amino acids, are not suitable to analyze with RPLC because they exhibit low hydrophobicity, which leads to weak interaction with stationary phase, poor retention and separation in RPLC mode^19^,^37^. Hydrophilic liquid interaction chromatography (HILIC) has been demonstrated to be a preferable technique for the chromatographic retention and separation of polar compounds^38^,^39^. Recently, a few studies indicated that HILIC could be also used to separate lipids according their polarity, and exhibited extraordinary separation efficiency^40^,^41^,^42^. We thus presume that HILIC can be optimized for concurrent analysis of polar metabolites and lipids in blood plasma samples.

The goal of this work is to develop a comprehensive approach based on HILIC for concurrently profiling of polar metabolites and lipids in human plasma. Moreover, high-resolution hybrid linear quadrupole ion trap-Fourier transform ion cyclotron resonance mass spectrometer (LTQ-FTMS), was chosen for the detection of metabolites. Standard mixture of polar metabolites and lipids was used for the development of HILIC-FTMS method. By examining a series of organic extraction solvents, a cocktail of MeOH/ACN/Acetone (1:1:1, v/v/v) was found to be the optimal to pair with HILIC-FTMS for concurrent analysis. This method was further explored for the discovery of smoking-related biomarkers in human blood plasma samples.

## Methods

### Chemicals and reagents

Methanol (Mass pure grade), acetonitrile (Mass pure grade), chloroform, acetone, formic acid, ammonium hydroxide and ammonium formate (10 M, pH 7.4) were from Fisher Scientific. Chemical standards were purchased from Sigma Aldrich (St. Louis, MO, USA) and Avanti Polar Lipids (Alabaster, AL). Standard stock solutions of 7 polar metabolites and 7 lipids were prepared for the development of concurrent profiling method on HILIC-FTMS. For polar metabolites, adenine (Ade), guanosine (Guo), L-Arginine (Arg), creatinine (Cr), L-histidine (His), L-phenylalanine (Phe) and sucrose (Suc) were selected as representatives of 4 important polar metabolite classes: neucleobase, neucloside, amino acid and sugar. Ade was dissolved in 50% methanol while other 6 polar metabolites were dissolved in water. For lipids, 1,3-Dipentadecanoin (DG), 17:0 (2S-OH) ceramide (Cer), 1-heptadecanoyl-2-hydroxy-sn-glycero-3-phosphocholine (LysoPC (17:0)), 1,2-diheptadecanoyl-sn-glycero-3-phosphocholine (PC (17:0/17:0)), 1,2-diheptadecanoyl-sn-glycero-3-phosphoethanolamine (PE (17:0/17:0)), 1,2-diheptadecanoyl-sn-glycero-3-phosphate (PA (17:0/17:0)) and 1,2-diheptadecanoyl-sn-glycero-3-phospho-(1’-rac-glycerol) (PG (17:0/17:0)), which represents 3 important lipid classes (Glycerolipid, Ceramide, Glycerophospholipid), were selected and dissolved in methanol. Water was purified by a Milli-Q Gradient ultrapure water purification system (Millipore, Billerica, MA). All other chemicals were of analytical grade.

### Lipid nomenclature

Lipids were named according to Lipid Maps (http://www.lipidmaps.org); *e.g.* 1,2-diheptadecanoyl-sn-glycero-3-phosphocholine is designated PC (17:0/17:0). When the fatty acid chain could not be determined, the total number of carbons and double bonds of all fatty acyl chains are given, *e.g.* PE (38:2).

### Blood sample collection

Human plasma samples were collected from 9 male volunteers (4 heavy smokers and 5 non-smokers) before breakfast. The age of volunteers ranges from 18 to 31. The median ages (IQR) of smokers and non-smokers are 26 and 25, respectively. The median BMI of smokers and non-smokers are 24.2 and 23.8, respectively. All of the volunteers are self-reported as healthy and haven’t taken any medications for at least 2 weeks before their blood samples were collected. The detailed criterions for the selection of voluntary participants are listed in Table S1. The fasting time is longer than 10 hours (overnight). Informed consent was obtained from all subjects. Experiments were performed in accordance with the National Institutes of Health Guidelines on the Human Subjects Research. All experimental protocols were approved by the biosafety committee of University of California Irvine. The fasting blood sample (30 mL) was added to a tube with Heparinum and centrifuged at 3000 rpm/min, 4 °C for 10 min. The supernatant was transferred to five 5 mL tubes and stored at −80 °C until assayed. Plasma samples were thawed on ice before extraction by various methods. The pooled plasma sample was prepared by pooling 500 μL of each of the 9 plasma samples. A blank sample prepared by replacing the plasma with pure water was used to assess contamination introduced during sample preparation.

### Preparation of mixed-standard sample

Mixed standards were prepared by dissolving stock of Ade, Guo, Arg, Cr, His, Phe, Suc, Cer, LysoPC, PC, PE, PA, PG and DG in 50% acetonitrile. The final concentrations of these 14 standards were listed in Table 1.

### Metabolite extraction by single, combined or biphasic solvents

100 μL aliquots of plasma sample were treated with 300 μL single or combined organic solvents including MeOH, MeOH/ACN/acetone (1:1:1, v/v/v), or a biophasic solvent of 1200 μL CHCl_3_/MeOH (2:1) and 400 μL H_2_O. Samples were vortexed and kept in −80 °C for 2 hours for a complete extraction and protein precipitation, followed by centrifugation at 13000 rpm/min, 4 °C for 10 min. The supernatants or CHCl_3_ layer was collected, dried under N_2_ and dissolved in 100 μL MeOH/water (1:1, v/v), and stored at −80 °C for further analysis.

### Liquid chromatography separation

HILIC and RP separations were performed on a Surveyor LC system coupled to a LTQ-FTMS, containing a heated electrospray ionization source (ESI) (Thermo Fisher Scientific, Waltham, MA). The column and auto-sampler temperatures were maintained at 25 °C and 4 °C, respectively. The injection volumes were 15 μL and 5 μL for standard mixture and plasma samples, respectively.

For HILIC separation, an Atlantis silica column (2.1 mm × 150 mm, 100 Å, 3 μm, Waters, Milford, MA) was used for HILIC separation. Acetonitrile and water modified with 50 mM ammonium formate were used as mobile phase A and B, respectively. The column was eluted with a liner gradient from 5–50% B over 20 min, a linear gradient to 5% B over 0.1 min, isocratic conditions at 5% B for 9.9 min, at a flow rate of 0.2 mL/min.

For RP separation, a Luna C5 column (2.1 × 100 mm, 100 Å, 5 μm, Phenomenex, Los Angeles, CA) and a Zorbax C18 column (2.1 × 100 mm, 100 Å, 3.5 μm, Agilent, Santa Clara, CA) were used for RP analysis. Mobile phase A was water: acetonitrile (98:2, v/v); mobile phase B was acetonitrile: water (35:65, v/v); and mobile phase C was isopropanol: acetonitrile: water (60:35:5, v/v/v). All of the mobile phases were modified with 50 mM ammonium formate. The C5 and C18 column were eluted with a gradient as follows: a liner gradient of 100–0% A and 0–100% B over 10 min; a liner gradient of 100–0% B and 0–100% C for 10 min; isocratic conditions at 100% C for 5 min; a linear gradient to 100% A, 0% B and 0% C over 0.1 min; isocratic conditions at 100% A for 5 min. The gradient duration was 30 min at a flow rate of 0.2 mL/min.

### Mass spectrometry detection

A Thermo Finnigan LTQ-FTMS (Thermo Fisher Scientific, Waltham, MA) was set to collect data from m/z 50 to 1200 in centroid mode. External calibration was carried out with a standard LTQ calibration mixture (Thermo Scientific, Waltham, MA). Following settings were used for MS detection: vaporizer temperature, 280 °C; sheath and auxiliary gases, 35 and 15 (arbitrary units); spray voltage, 3.5 kV; capillary temperature, 350 °C; capillary voltage, 10 V; tube-lens voltage, 120 V; maximum injection time, 1000 ms; maximum number of ions collected for each scan, 5 × 10^5^; mass resolution, 10^5^.

### Data analysis

Data were collected continuously over the 30 min chromatographic separation. In order to compare the detected features of different extraction methods, Xcalibur file converter software (Thermo Fisher, San Diego, CA) was used to convert the raw data to cdf files for further data processing in R project. An adaptive processing software package (apLCMS, http://www.sph.emory.edu/apLCMS)^43^ designed for LC-FTMS data was used for peak extraction. This software obtained m/z feature tables through 5 major processing steps: (1) noise filter, (2) peak identification by peak location (m/z and retention time), peak width and intensity, (3) retention time correction, (4) m/z peak alignment across multiple spectra, and (5) re-analysis to capture peaks originally missed because of weak signal relative to the signal-to-noise filter. Regarding the metabolite recovery, 42 metabolites with varying polarities were selected to compare their base-10 log-transformed peak areas. The peak area was calibrated by following formula: 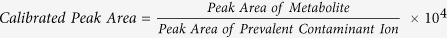, where the prevalent contaminant ion with a m/z of 427.39118 (RT = 14.5 min) was detected in all the blanks and samples.

The raw data of samples from heavy smokers and non-smokers were converted to mzXML data format using proteoWizard software (Spielberg Family Center for Applied Proteomics, Los Angeles, CA) for further data processing. Peak detection, retention time collection and alignment were processed on the XCMS platform (https://metlin.scripps.edu/xcms/)^44^. All data-collection parameters were set to the “HPLC Orbitrap” default values except the following: maximal tolerated m/z deviation in consecutive scans = 3.5 ppm; width of overlapping m/z slices (mzwid) = 0.005; retention time window (bw) = 30 (seconds). Lists of retention times (RT), m/z values and peak intensities were exported to an Excel spreadsheet for processing. Preprocessed data sets were analyzed using Matlab (MathWorks, Natick, MA) and Metaboanalyst 3.0 (www.metaboanalyst.ca)^45^ to perform scatter plot, heat map, cluster analysis and partial least squares discriminant analysis (PLS-DA). The fold changes and p-values of student T-test were calculated in excel 2010. Features in MeOH/ACN/Acetone extracts and the significant features for cigarette smoke were searched against the METLIN^46^ and the Human Metabolome Database (HMDB)^47^ with the mass accuracy of 10 parts per million to identify putative metabolites.

## Results and Discussion

### Using mixed standards to establish HILIC-FTMS analysis method

HILIC has been popularly used to separate polar compounds on polar stationary phases such as silica, diol, amino, amide, and Zwitter ionic columns^48^. In addition, silica and diol columns have exhibited successful separation to lipids according to their polarity^40^,^49^. A silica column was selected for concurrent separation of lipids and polar metabolites in this study. Ammonium formate was added in mobile phase to improve separation efficiency. High efficient separation of lipids was expected because of the secondary interactions, such as hydrogen bonding and electrostatic interaction, between the polar stationary phase and lipids.

**Figure 1:**
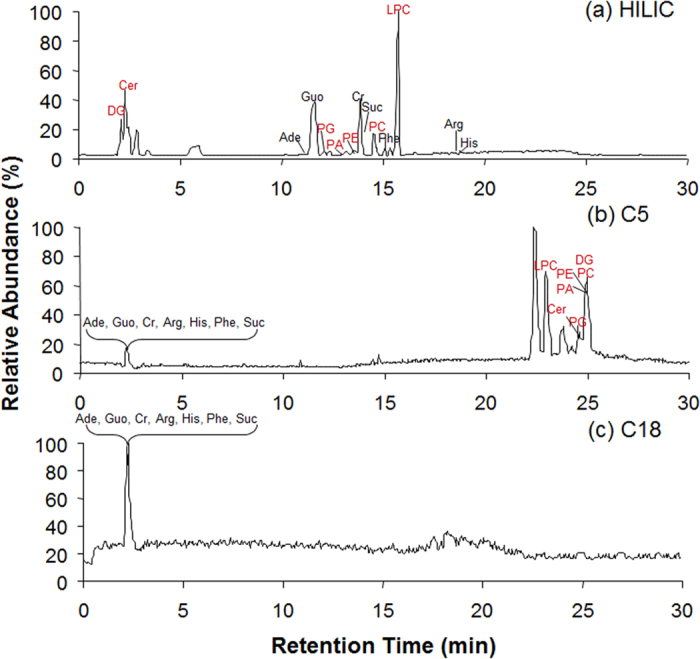
Total ion chromatograms of the standard mixture of 7 polar metabolites and 7 lipids on (**a**) Silica HILIC, (**b**) C5 and (**c**) C18. Ade: adenine; Guo: guanosine; Cr: creatinine; Arg: L-Arginine; His: L-histidine; Phe: L-phenylalanine; Suc: sucrose; LPC: LPC (17:0); PC: PC (17:0/17:0); PE: PE (17:0/17:0); PA: PA (17:0/17:0); PG: PG (17:0/17:0); Cer: Cer (d18:1/17:0(2S-OH)); DG: DG (15:0/15:0).

The HILIC separation conditions were optimized and evaluated by 14 standards including 7 polar metabolites and 7 lipids (Table 1). As shown in Fig. 1, the silica HILIC column showed much higher separation efficiency for the mixed standards, compared to popularly used RPLC columns (C5 and C18). The retention times of the 14 standards on the three columns were listed in Table 1. On C5 or C18 columns, all the polar metabolites were co-eluted in one chromatographic peak almost in the dead time even though 98% water was used as starting gradient, suggesting a poor retention for these compounds on RP columns. Contrarily, the polar metabolites were well retained and separated on the silica column in HILIC mode, with retention times ranging from 10 to 20 min. For lipids, although they could be separated in the RPLC mode according to their hydrophobicity (LysoPC, Cer, PG, PA, PE, PC, DG), the separation is time consuming, especially for C18 column, from which the lipids can not be eluted in 30 min. In contrast to their strong retention on RP columns, the 7 lipids could be well separated on silica HILIC column within 20 minutes in order of polarity: DG, Cer, PG, PA, PE, PC, LysoPC. As expected, the polar lipids were retained based on their polar active groups, while the secondary interactions between HILIC stationary phase and lipids allowed the weak retained low-polar lipids to be separated based on their hydrophobicity (carbon chain length) and unsaturation (number of C=C bonds). Considering both the separation resolution and efficiency, silica HILIC-FTMS is the best method for concurrent analysis of polar metabolites and lipids. The deviations of retention and peak areas, and mass accuracy of the HILIC-FTMS method was tested by a 6-run sequence of the standards. The coefficients of variations (CVs) of RTs were all lower than 3.43%, the CVs of peak areas were all lower than 10.36%, and the mass accuracies were less than 3.5 ppm with an average level of 2.3 ppm (Table 2).

### Developing a cocktail of MeOH/ACN/Acetone for metabolite extraction in plasma samples

**Figure 2:**
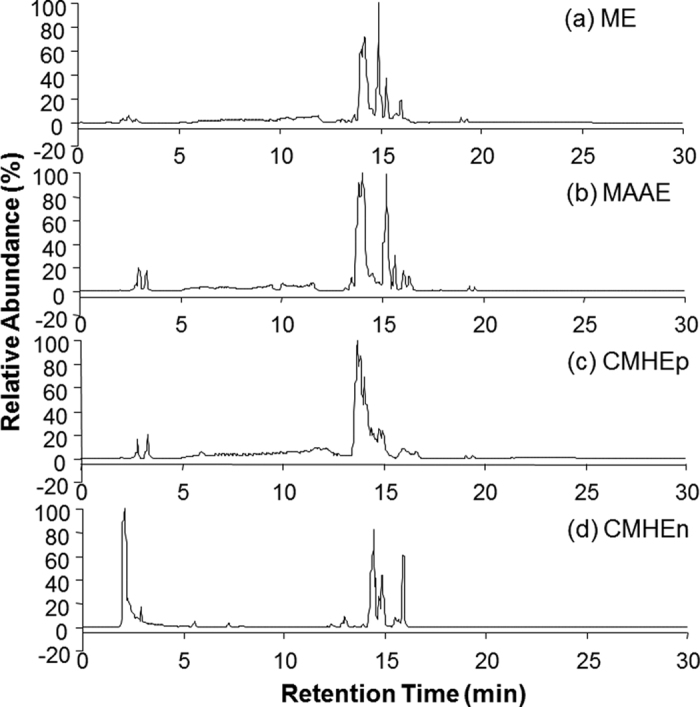
Base peak chromatograms (BPCs) for the four extracts: (**a**) methanol extract (ME), (**b**) MeOH/ACN/Acetone extract (MAAE), (**c**) polar fraction of CHCl_3_/MeOH/H_2_O extraction (CMHEp) and (**d**) non polar fraction of CHCl_3_/MeOH/H_2_O extraction (CMHEn), in positive ionization mode.

In order to find an optimal cocktail of extraction solvents, we examined the extraction efficiencies of methanol, MeOH/ACN/Acetone (1:1:1, v/v/v) and CHCl_3_/MeOH/H_2_O (2:1:1, v/v/v) as representatives of single, combined and biphasic extraction solvents, respectively. Single and combined extraction solvents can extract both polar and non-polar metabolites in one single phase, denoted as methanol extract (ME) and MeOH/ACN/Acetone extract (MAAE), respectively, while biphasic solvent, CHCl_3_/MeOH/H_2_O extraction (CMHE) will generate two fractions including polar fraction (CMHEp) and non polar fraction (CMHEn). To compare the efficiency of these extraction methods, the extracts were analyzed on HILIC-FTMS. The base peak chromatograms (BPCs) of the extracts in positive ionization mode are shown in Fig. 2. The BPC chromatogram of ME (Fig. 2a) is very similar to that of the MAAE (Fig. 2b). As expected, the BPC chromatograms of CHCl_3_/MeOH/H_2_O extracts, CMHEp and CMHEn, are very different. The unretained peak of CMHEn fraction at 2 min is much larger than that of the ME, MAAE and CMHEp, while the latter three fractions have a bigger peak around 13.7 min. In addition, the peaks eluted between 14–16 min are much less in the CMHEp fraction than the other extracts. Similar results were obtained in negative mode. Although we can not determine the best extraction approaches based on BPC results, ME and MAAE methods are better than CMHE from the perspective of analysis efficiency because it’s more time consuming to perform a parallel analysis of the two CMHE fractions.

**Figure 3:**
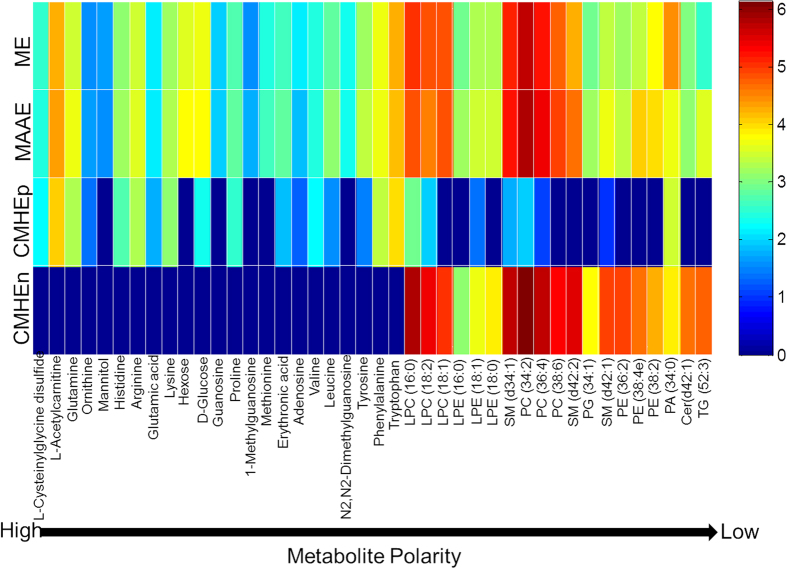
Comparison of the metabolite recovery of ME, MAAE and CMHE. The peak areas of the 42 metabolites were normalized to the peak area of an ion (m/z = 427.39118, RT = 14.5 min) which could be detected in all the blanks and samples, and then multiplied by 10,000, followed by a base-10 log-transformation. Mean values from replicates were used for comparison.

To further evaluate the extraction methods, the LC-FTMS data from ME, MAAE and CMHE extracts were processed by apLCMS^43^ to obtain feature lists including m/z, RT and ion intensities. For all the extracts, more features were detected in the ESI + mode compared to the ESI- mode. Table 3 shows the numbers of features detected in all replicates from both the positive (ESI+) and negative (ESI−) LC-FTMS. In total, 1443, 1825 and 1741 (881 features in non polar fraction plus 860 features in polar fraction) features were detected in ME, MAAE and CMHE, respectively. In respect of the numbers of detected features, MAAE is the best. Regarding the metabolite recovery, 42 metabolites with varying polarities were selected to compare their peak areas, which were calibrated by the peak area of a prevalent contaminant ion (m/z = 427.39118, RT = 14.5 min) which had been detected in all the blanks and samples, and then multiplied by 10,000, followed by a base-10 log-transformation^50^. As shown in Fig. 3, ME and MAAE had comparable recoveries for vast majority of those metabolite features with high or medium polarity, while MAAE shown better recoveries for several low polarity lipids including sphingomyelin (SM (d42:2)), PG (34:1), SM (d42:1), PE (36:2), PE (38:4e), PE (38:2) and triglyceride (TG (52:3)). Although CMHE exhibited better recoveries of nonpolar metabolites/lipids, it showed poor recoveries for polar metabolites and some of low-level polar metabolites were even not detected in the CMHEp fraction. Thus, the CMHE method is biased for the extraction of nonpolar metabolites/lipids. In terms of sample throughput, numbers of detected metabolite features and extraction recoveries, MAAE is the best compared to ME and CMHE.

### Concurrent profiling of polar metabolites and lipids in plasma samples from heavy smokers and non-smokers

**Figure 4:**
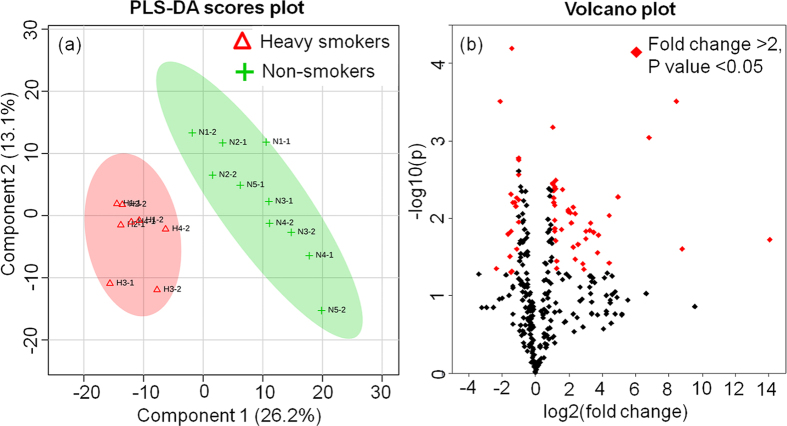
(**a**) PLS-DA scores plots for comparison of the global metabolite profiles in (∆) heavy smokers and (+) non-smokers. 26.2% and 13.1% are the scores of the component 1 and 2, respectively, in the PLS-DA analysis. (**b**) Volcano plot of the 294 features with VIP values > 1 in PLS-DA analysis. Volcano plot is a combination of fold change and t-tests. The 62 features with a fold change >2 and p-value < 0.05 were marked in red.

**Figure 5:**
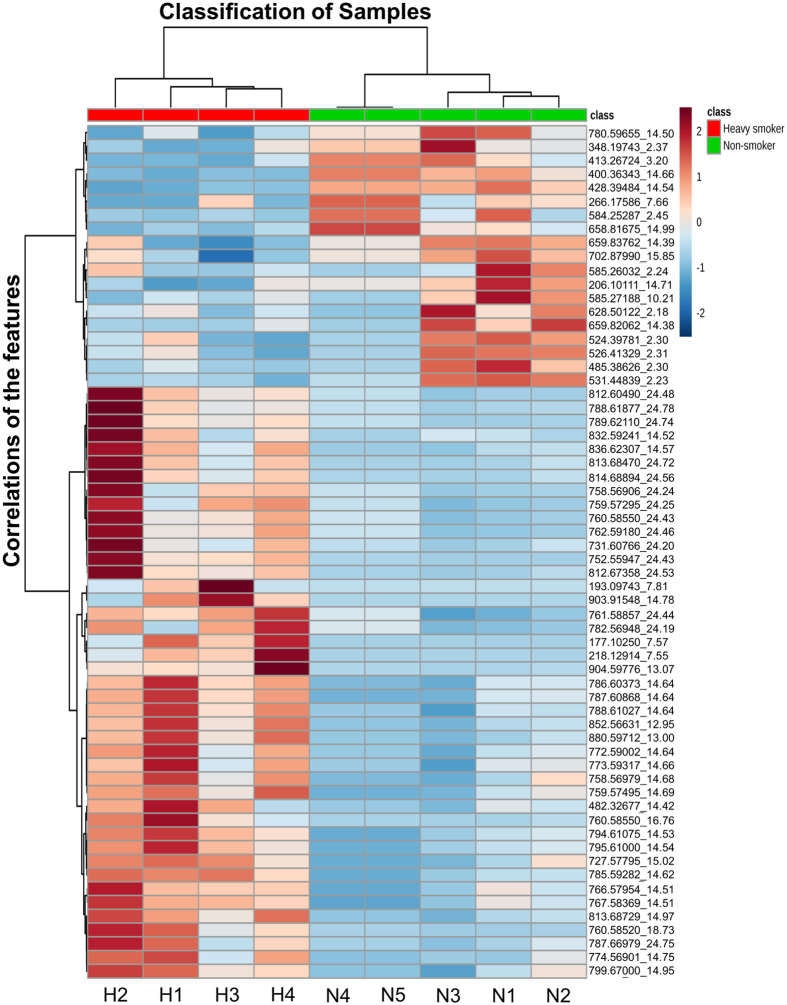
The levels and correlations of the 62 significant features in smokers and non-smokers. The log-transformed normalized intensities of the 62 features in the 9 samples were shown in the heat map. Obviously, 43 features were elevated in heavy smokers when compared to non-smokers while 19 features were reduced in the former. The correlations of the 62 features were shown by the Hierarchical clustering dendrogram. H1-H4: heavy smokers; N1-N5: non-smokers.

The developed method was applied to analyze plasma samples collected from heavy smokers because tobacco smoking causes a variety of diseases due, in large part, to oxidative stress as well as multitude of metabolic changes that are poorly understood. Herein, for a proof-of-concept test of clinical use of our concurrent analysis approach, a small number of plasma samples from 4 male heavy smokers (>30 cigarettes per day) and 5 male non-smokers were analyzed to assess the impacts of cigarette smoke on smokers’ global metabolite profiles. Partial least squares discriminant analysis (PLS-DA) was used to perform supervised classification (Fig. 4a) and feature selection, top 294 features with a variable importance in the projection (VIP) value > 1 were kept for further study. Cross validation was used to evaluate and optimize the PLS-DA model, and our model showed an accuracy of 94.4% and a Q2 value of 71.7%, suggesting it has good predictive ability. The heavy smokers could be clearly discriminated from the global metabolite profiles by our concurrent analysis approach. Furthermore, we examined the significantly changed features from the 294 features by Volcano plot (Fig. 4b), which is a combination of fold change and t-tests. Among them, 62 significantly changed features with fold changes >2 and p values < 0.05 were successfully identified, suggesting that Volcano plot is an appropriate method to identify the significantly changed features from the metabolites of human plasma by HILIC-FTMS analysis. These results demonstrated that our concurrent analysis method could be potentially used for biomarker discovery in human plasma samples. The significantly changed features were incorporated into a heat map to visualize their levels in smokers’ and non-smokers’ group as well as the correlations among different features (Fig. 5). The smokers’ and non-smokers’ plasma samples were labeled with red and green ribbons, respectively. The heat map showed that among the 62 smoking-related biomarker candidates, 43 features were elevated and 19 features were reduced in heavy smokers compared to non-smokers. The mass data (m/z) that could be annotated with database such as HMDB, KEGG were listed on the left side of the figure. The distance between each two features on the right side represents their correlation. For instance, the short distance between feature 1 (m/z: 177.10250) and feature 2 (m/z:193.09743) suggests a high correlation. This is well supported by m/z database search result, showing that feature 1 and feature 2 should be cotinine and hydrocotinine, respectively, and both of them are important metabolites of nicotine. The correlation information of these identified metabolites may benefit the studies on smoking-related AOPs.

Although this study did not perform in-depth research on the identification of the significant features because of the limitations on sample size and putative structures, some information of the significant features could be obtained according their accurate mass by searching literatures, the list of 1183 nonionic endogenous chemicals (supplementary information Dataset 1) and the HMDB. Among the 62 candidate smoking-related biomarkers, we identified the potential structures of 25 features (supplementary information Table S2), including 5 polar metabolites and 20 lipid features. As shown in Table S2, the polar metabolites are cotinine, hydroxycotinine, Arginyl-Valine, 2-Acetamido-2,6-dideoxy-D-glucose and bilirubin. The 20 lipid features are phospholipids or sphingomyelin. The mass differences between these observed features and their putative metabolites were all less than 10 ppm. The levels of phospholipids and sphingomyelin were elevated in heavy smokers (Fig. 5 and Table S2), consistent with the results that have been reported in a targeted study of phospholipids and sphingomyelins in serum samples from smokers and non-smokers^51^. Since cigarette smoke could directly interact with epithelial cell in the lung and result in membrane damage^52^, there are substantial reports showing that phospholipid (a major component of biological membrane) metabolism and degradation pathways can be activated by cigarette smoke^53^. Our data indicates that a high phospholipid turnover may be needed in the blood of smokers in response to the membrane damage induced by cigarette smoke. The classification and identification results showed the potential of our comprehensive metabolite profiling approach to concurrently analyze the polar metabolites and lipids in smokers, and to identify smoking-related biomarkers. The application of this method to a larger sample set merits further study.

In summary, a HILIC-FTMS based concurrent analysis method was established in this study to examine polar metabolites and lipids in human plasma samples. We demonstrated that HILIC has much better separation efficiency for 14 standards than conventional RP methods. A cocktail of MeOH/ACN/Acetone (1:1:1, v/v/v) was found to show high efficiency for the extraction of both polar metabolites and lipids from human plasma. This extraction method exhibits comprehensive coverage and good recoveries for the metabolites while minimum sample handing and less time consuming. Furthermore, the extraction cocktail of MeOH/ACN/Acetone paired with HILIC-FTMS was used for the analysis of human plasma samples. Heavy smokers could be successfully discriminated from non-smokers by PLS-DA classification of the metabolic profiling data. Further statistical analysis indicated that 62 features were significantly changed in heavy smokers compared to non-smokers. Although the identification of potential biomarker was not carried out in-depth in this study, the identification of candidate metabolites such as cotinine, hydroxycotinine, Arginyl-Valine, LysoPE (18:0), PC (36:2) and SM (d42:2), was intriguing. The concurrent analysis approach developed in this study could be explored for biomarker discovery and metabolite-based diagnosis.

## Additional Information

**How to cite this article**: Cai, X. and Li, R. Concurrent profiling of polar metabolites and lipids in human plasma using HILIC-FTMS. *Sci. Rep.* **6**, 36490; doi: 10.1038/srep36490 (2016).

**Publisher’s note**: Springer Nature remains neutral with regard to jurisdictional claims in published maps and institutional affiliations.

## Supplementary Material

Supplementary Information

Supplementary Dataset 1

## Figures and Tables

**Table 1:** Retention times of the 14 standards.

Standard	Class	Concentration (μM)	Formula	m/z	RTs (min)		
					HILIC	C5	C18
DG	Glycerolipid	10	C_33_H_64_O_5_	558.50972	2.16	24.95	n/a
Cer	Ceramide	2	C_35_H_69_NO_4_	568.53046	2.31	24.58	n/a
Ade	Nucleobase	40	C_5_H_5_N_5_	136.06232	11.17	2.32	2.16
Guo	Nucleoside	40	C_10_H_13_N_5_O_5_	284.09949	11.58	2.27	2.16
PG	GlyceroPhospholipid	10	C_40_H_78_O_10_P	768.57542	12.08	24.58	n/a
PA	GlyceroPhospholipid	10	C_37_H_73_O_8_P	694.53865	12.82	24.86	n/a
PE	GlyceroPhospholipid	10	C_39_H_78_NO_8_P	720.55430	13.50	24.86	n/a
Cr	Amino acid	40	C_4_H_7_N_3_O	114.06673	13.90	2.27	2.16
Suc	Sugar	40	C_12_H_22_O_11_	360.15058	14.09	2.27	2.05
PC	GlyceroPhospholipid	10	C_42_H_84_NO_8_P	762.60125	14.71	24.95	n/a
Phe	Amino acid	40	C_9_H_11_NO_2_	166.08680	15.03	2.32	2.26
LysoPC	GlyceroPhospholipid	10	C_25_H_52_NO_7_P	510.35595	15.72	22.94	n/a
Arg	Amino acid	40	C_6_H_14_N_4_O_2_	175.11950	18.75	2.23	2.05
His	Amino acid	40	C_6_H_9_N_3_O_2_	156.07730	18.97	2.23	2.05

**Table 2:** Deviations of retention times (RTs), peak areas, and mass accuracy of the 14 compounds in the standard mixture.

Standard	*m/z*	RTs (min)		Delta (ppm)			Peak Area	
		mean	CV (%)	mean	min	max	mean	CV (%)
Adenine	136.06232	11.17	0.87	2.92	2.79	2.94	639646.70	1.62
Guanosine	284.09949	11.58	0.63	1.53	1.41	1.62	4535286.00	3.89
L-Arginine	175.11950	18.75	0.28	2.54	2.51	2.57	304883.30	8.90
Creatinine	114.06673	13.90	0.61	3.33	3.24	3.51	31382.33	2.80
L-histidine	156.07730	18.97	0.39	2.55	2.50	2.56	114596.00	10.34
L-phenylalanine	166.08680	15.03	0.35	2.66	2.59	2.71	274661.70	5.02
Sucrose	360.15058	14.09	0.58	0.86	0.69	1.03	221366.00	8.37
Cer (d18:1/17:0 (2S-OH))	568.53046	2.31	1.53	0.82	0.07	1.51	4580576.00	4.54
LPC (17:0)	510.35595	15.72	0.44	2.96	2.66	3.25	5543540.00	2.72
PC (17:0/17:0)	762.60125	14.71	0.37	3.20	2.95	3.44	1794670.00	8.48
PE (17:0/17:0)	720.55430	13.50	0.51	2.75	2.22	3.50	807101.70	7.72
PA (17:0/17:0)	694.53865	12.82	0.68	1.21	1.02	1.55	843154.00	8.03
PG (17:0/17:0)	768.57542	12.08	0.77	2.32	1.86	3.06	1777743.00	10.36
DG (15:0/15:0)	558.50972	2.16	3.43	2.62	1.50	3.47	15384.33	0.61

**Table 3:** Numbers of the features extracted by apLCMS.

	ME	MAAE	CMHEn	CMHEp
ESI+	1000	1286	549	527
ESI−	443	539	332	333
Total	1443	1825	881	860
