# Supplementary material for: Concurrent profiling of polar metabolites and lipids in human plasma using HILIC-FTMS
Source: Sci Rep. 2016 Nov 7;6:36490. doi: 10.1038/srep36490 (PMC5098236; doi:10.1038/srep36490)
Supplement: Supplementary Information [file srep36490-s1.pdf]

Supplementary Information:

Concurrent profiling of polar metabolites and lipids in human plasma using HILIC-FTMS

Xiaoming Cai[ǂ,₸], Ruibin Li[˧,┴,]*

[ǂ] College of Pharmaceutical Science, Soochow University, Suzhou, 215123, China;
[˧] School for Radiological and Interdisciplinary Sciences (RAD-X), Collaborative Innovation Center of Radiation Medicine of Jiangsu Higher Education Institutions, Soochow University, Suzhou 215123, China;
[₸] Department of Pharmacology, University of California, Irvine, Irvine, CA 92697, United States;
[┴] Division of NanoMedicine, Department of Medicine, University of California, Los Angeles, CA 90095, United States

*Corresponding author: Ruibin Li, Ph.D.
Department of Medicine, Division of NanoMedicine, UCLA School of Medicine, 52-175 CHS, 10833 Le Conte Ave, Los Angeles, CA 90095-1680.

Tel: (310) 983-3359, Fax: (310) 206-8107

E-mail: liruibin@ucla.edu

Tables

Table S1. Criterions for the selection of voluntary participants

Characteristics	Smokers	Non-smokers
Age	18-31	18-31
Gender	Male	Male
BMI (kg/m^2)	18-25	18-25
Race	White American	White American
Medical history	Healthy	Healthy
Consumption of cigarette	≥30 per day	0
Medication usage	No medication for 2 weeks	No medication for 2 weeks
Exercise	5-10 hours per week	5-10 hours per week
Consumption of vegetables	2-5 kg per week	2-5 kg per week
Consumption of Meat	1-3 kg per week	1-3 kg per week
Consumption of Fish	0.1-1 kg per week	0.1-1 kg per week

Table S2. Potential structures of candidate smoking-related biomarkers

m/z	RT (min)	Somker *vs* non-smoker	Potential structure	Mass difference (ppm)
177.10250	7.57	↑	Cotinine	1.47
193.09743	7.81	↑	Hydroxycotinine	1.43
206.10111	14.71	↓	2-Acetamido-2,6-dideoxy-D-glucose	5.77
256.17586	7.66	↓	Arginyl-Valine	5.78
482.32677	14.42	↑	LysoPE (18:0)	5.54
585.27188	10.21	↓	Bilirubin	2.03
758.56979	14.68	↑	PC (34:2)	0.47
759.57495	14.69	↑	Isotope of PC (34:2)	N/A
760.58520	18.73	↑	PC (34:1)	0.16
760.58550	16.76	↑	Isomer of PC (34:1)	0.55
766.57954	14.51	↑	PC (O-36:5)	6.55
767.58369	14.51	↑	Isotope of PC (O-36:5)	N/A
772.59002	14.64	↑	PE (38:2)	6.39
773.59317	14.66	↑	Isotope of PE (38:2)	N/A
786.60373	14.64	↑	PC (36:2)	3.81
787.60868	14.64	↑	Isotope of PC (36:2)	N/A
788.61027	14.64	↑	Isotope of PC (36:2)	N/A
794.61075	14.53	↑	PC (O-38:5)	6.21
795.61000	14.54	↑	Isotope of PC (O-38:5)	N/A
813.68729	14.97	↑	SM(d42:2)	3.55
832.59241	14.52	↑	PC (40:7)	8.80
836.62307	14.57	↑	PC (40:5)	8.00
852.56631	12.95	↑	PI (34:2)	7.81
880.59712	13.00	↑	PI (36:2)	7.00
904.59776	13.07	↑	PI (38:4)	7.53

Figures

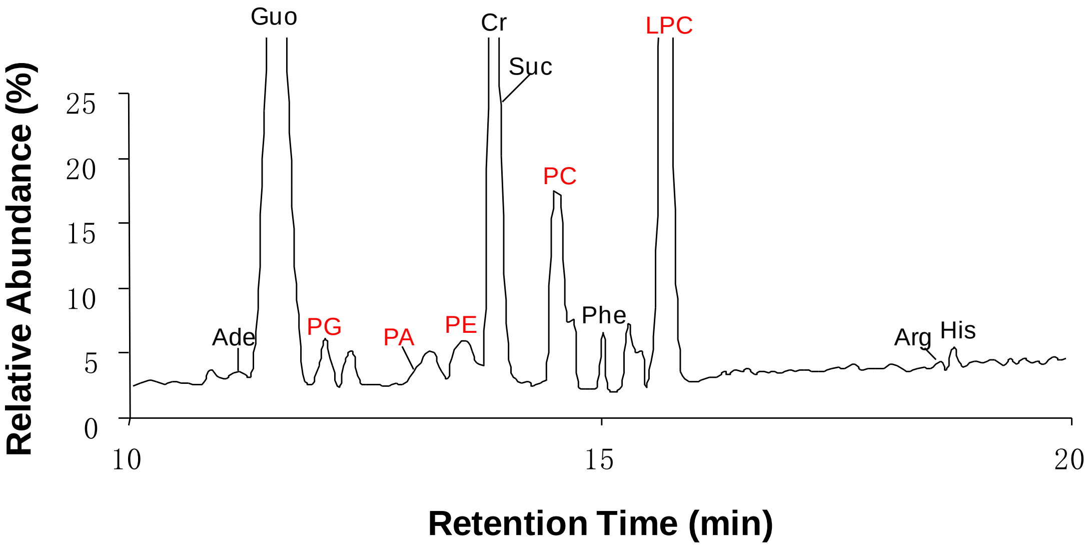

Figure S1 Amplified total ion chromatograms (from 10 min to 20 min) of the standard mixture of 7 polar metabolites and 5 polar lipids on Silica HILIC. Ade: adenine; Guo: guanosine; Cr: creatinine; Arg: L-Arginine ; His: L-histidine; Phe: L-phenylalanine; Suc: sucrose; LPC: LPC (17:0); PC: PC (17:0/17:0); PE: PE (17:0/17:0); PA: PA (17:0/17:0); PG: PG (17:0/17:0).
